# Assessment of Silicone Oil Emulsification: A Comparison of Currently Applied Methods

**DOI:** 10.1155/2023/8114530

**Published:** 2023-04-24

**Authors:** Hongmei Zhao, Qian Chen, Jian Yu, Kaicheng Wu, Yuan Zong, Chunhui Jiang, Haohao Zhu, Gezhi Xu

**Affiliations:** ^1^Department of Ophthalmology and Vision Science, Eye and ENT Hospital, Fudan University, Shanghai 200031, China; ^2^Key Laboratory of Myopia of State Health Ministry, Key Laboratory of Visual Impairment and Restoration of Shanghai, Shanghai 200031, China; ^3^NHC Key Laboratory of Myopia (Fudan University), Key Laboratory of Myopia, Chinese Academy of Medical Sciences, Shanghai 200031, China; ^4^Department of Ophthalmology, Shanghai Fifth People's Hospital, Fudan University, Shanghai 200240, China

## Abstract

**Purpose:**

To compare ultrasound biomicroscopy (UBM), Coulter counter, and B-scan ultrasonography in the evaluation of silicone oil (SO) emulsification.

**Methods:**

Patients who underwent primary pars plana vitrectomy with SO tamponade for rhegmatogenous retinal detachment and SO removal were included. UBM images were acquired before the SO removal, and B-scan images were taken after removal. The number of droplets in the first and last 2 mL of washout fluid was analyzed using a Coulter counter. The correlations between these measurements were analyzed.

**Results:**

Thirty-four eyes received both UBM and Coulter counter analysis for the first 2 mL of washout fluid, and 34 underwent B-scan and Coulter counter analysis of the last 2 mL washout fluid. The mean UBM grading was 26.41 ± 9.71 (range: 1–36); the mean SO index obtained with B-scan was 5.25 ± 5.00% (range: 0.10–16.49%), and the mean number of SO droplets was 1.26 ± 2.45 × 10^7^/mL and 3.34 ± 4.22 × 10^6^/mL in the first and last 2 mL of washout fluid, respectively. There were significant correlations between UBM grading and SO droplets in the first 2 mL and between B-scan grading and SO droplets in the last 2 mL (all *P* < 0.05).

**Conclusions:**

UBM, Coulter counter, and B-scan ultrasonography could all be used in the evaluation of SO emulsification, and their findings were comparable.

## 1. Introduction

Silicone oil (SO), first introduced by Cibis et al. in the 1960s [1], is now widely used in the management of complicated retinal detachment. However, complications from this procedure have also been reported. The most frequent is SO emulsification, which is correlated with several other pathological findings, including glaucoma [2], inflammation, proliferation [3], cataract [4], and keratopathy [5], and possibly causes them. In addition, emulsified SO droplets were reported within the retina [6–8], optic nerve [9, 10], and even in extraocular structures [11, 12].

Several methods have been proposed to detect and evaluate SO emulsification, including slit lamp [13], ophthalmoscopy, gonioscopy [14], phase-contrast microscopy [15], ultrasound biomicroscopy (UBM) [16], B-scan ultrasonography [17, 18], and Coulter counter [19, 20]. Using gonioscopy and slit lamp, SO emulsification was observed in 83% [14] to 100% [13] of cases; furthermore, the diameter of emulsified SO droplets was evaluated with phase-contrast microscopy [15] and Coulter counter [19, 21].

Several studies had similar findings, and the close relationship between SO emulsification and elevated intraocular pressure (IOP) was confirmed by researchers using UBM [16] or Coulter counter [21]. However, some differences also emerged. For example, fluid-air exchange was found to be ineffective to reduce residual SO droplets in a study using B-scan ultrasonography [18] and effective in a study using a Coulter counter [20]. Slit lamp, UBM, and gonioscopy focus on the anterior segment, whereas B-scan ultrasonography focuses on the posterior one; thus, their results might differ. Moreover, slit lamp, UBM, and gonioscopy can evaluate SO emulsification both before and after SO removal, whereas B-scan ultrasonography can be used only after SO removal.

Thus, each of these studies only evaluated a piece of the puzzle, and the combination of different modalities can help us acquire a comprehensive understanding of SO emulsification. However, the measurements by different methods should be consistent to be combined. We compared UBM, Coulter counters, and B-scan ultrasonography results for the evaluation of SO emulsification in a group of patients who underwent vitrectomy and SO tamponade for rhegmatogenous retinal detachment (RRD). The relationship between these measurements was then analyzed.

## 2. Materials and Methods

### 2.1. Study Participants and Ethics Statement

This single-center study followed an observational, cross-sectional design. We collected data from patients who underwent primary pars plana vitrectomy (PPV) with SO tamponade for RRD at the Eye and ENT Hospital of Fudan University between January 2019 and January 2022. Patients who completed a minimum of 8-week follow-up after SO removal with the retina properly attached were eligible. Exclusion criteria were diabetes mellitus, previous SO injection, intraocular surgery other than PPV or cataract surgery, intraocular diseases other than RRD or cataract (e.g., glaucoma, uveitis), elevated IOP (>21 mmHg) before PPV, or age <18 years at the time of primary PPV.

The study was approved by the Institutional Review Board of the Eye and ENT Hospital of Fudan University and conformed to the tenets of the Declaration of Helsinki. All patients provided written informed consent. Patient who underwent both UBM examination before SO removal and Coulter analysis of the first 2 mL washout fluid were enrolled in the first group to study the possible correlations between the results of Coulter counter and the UBM grade. Patients who underwent both B-scan ultrasonography after SO removal and Coulter analysis of the last 2 mL washout fluid were enrolled in the second group to study the correlations between the results of the Coulter counter and SOI.

### 2.2. Surgical Procedures

During the SO injection surgeries, a standard three-port, 23-gauge PPV was performed in all patients by a single surgeon (Chunhui Jiang) using the Alcon Constellation system (Alcon Laboratories, Inc., Geneva, Switzerland) and SO (5700 cSt; Bausch & Lomb Inc., Bridgewater, NJ, USA). During SO removal, additional procedures, such as membrane peeling, phacoemulsification, and/or intraocular lens implantation, were performed as necessary. Constant irrigation of the vitreous cavity for 10 minutes was adopted to ensure thorough removal of the emulsified SO droplets.

### 2.3. Main Ophthalmic Measurements

Before and after SO removal, each patient underwent a thorough ophthalmic examination, which included the assessment of the best-corrected visual acuity, logarithm of the minimum angle of resolution (logMAR), spherical equivalent power (calculated as half of the cylindrical dioptric plus the spherical diopter), slit-lamp microscopy, dilated fundus examination with a non-contact lens (MaxField 84 Diopter; Ocular Instruments, Bellevue, WA, USA), and measurement of IOP by non-contact tonometry. The axial length (AL) measurement by IOLMaster (version 3.01; Carl Zeiss Meditec, Jena, Germany) was performed only preoperatively. Demographic data and clinical histories were also collected.

### 2.4. Silicone Oil Emulsification Examinations

The UBM examination of the anterior segment (MD-300L, 50-MHz probe transducer; Meda Co., Ltd., Tianjin, China) was performed within the week before SO removal. Washout fluid samples were collected during the SO removal for the Coulter counter analysis; then, the B-scan ultrasonography (AVISO, Quantel Medical, France) was performed 8–12 weeks postoperatively, when the inflammation or hemorrhage caused by SO removal had subsided.

### 2.5. Ultrasound Biomicroscopy Grading

The UBM exam and grading were performed according to the method previously described in detail [16, 22]. In brief, eight signs of SO emulsification in the UBM images were graded as 1 (present) or 0 (not present), and the grades for all signs in each eye were summed. All UBM images were analyzed by two independent readers (Hongmei Zhao and Jian Yu). When the grades determined by both graders were identical, they were used as the final grades; when they differed, the final grade was determined by a senior specialist (Qian Chen).

### 2.6. Coulter Counter

The number of SO droplets in the washout fluid samples was assessed with a Coulter counter, using the method introduced by Chan [19, 23]. In brief, the first and last 2 mL of washout fluid extracted during the SO removal were collected. Using a Multisizer® 3 Coulter counter (Beckman Coulter, Brea, CA, USA), the size and number of droplets in the samples were measured, and the particles ranging in diameter between 0.4 and 12 *μ*m were included in the analysis. The values for each sample represent the mean of three consecutive measurements. Based on the diameter, the droplets were divided into smaller (0.4–5 *μ*m) and larger (5–12 *μ*m) ones.

### 2.7. B-Scan Ultrasonography

Finally, B-scan ultrasonography examination and analysis were performed according to the methods previously described [17, 23]. The SO index (SOI) was calculated as the percentage of signals from hyperechoic droplets in the area of the vitreous cavity.

### 2.8. Statistical Analysis

All analyses were performed using SPSS software version 20.0 (IBM, Armonk, NY, USA). The Kolmogorov–Smirnov test was used to examine the normality of the data. Spearman's correlation coefficient was used to assess the correlations between the results of the Coulter counter and the UBM grade or SOI. *P* values <0.05 were considered statistically significant.

## 3. Results

Thirty-four patients underwent both UBM examination before SO removal and Coulter analysis of the first 2 mL washout fluid. Their mean age was 55.32 ± 13.78 years (range: 24–86); the mean duration of SO in situ was 28.11 ± 16.24 weeks (range: 14.00–103.57), and the mean AL was 25.63 ± 2.57 mm (range: 21.61–33.16). Five patients had choroidal detachment and four patients had a history of ophthalmic trauma at the time of PPV.

Another 34 patients underwent both B-scan ultrasonography after SO removal and Coulter analysis of the last 2 mL of washout fluid. Their mean age was 51.91 ± 16.76 years (range: 18–86); the mean duration of SO in situ was 27.46 ± 9.67 weeks (range: 14.86–55.00), and the mean AL was 25.79 ± 2.86 mm (range: 22.01–33.00). Two patients had choroidal detachment and six patients had a history of ophthalmic trauma at the time of PPV.

Emulsified SO was identified in all cases (100%) using the three methods: UBM, B-scan ultrasonography, and Coulter exam. The mean UBM grade of SO emulsification was 26.41 ± 9.71 (range: 1–36), and the mean SOI from B-scan ultrasonography was 5.25 ± 5.00% (range: 0.10–16.49%). The mean number of SO droplets measured with the Coulter counter was 1.26 ± 2.45 × 10^7^/mL (range: 0.10–12.26 × 10^7^/mL) and 3.34 ± 4.22 × 10^6^/mL (range: 0.46–21.89 × 10^6^/mL) in the first and last 2 mL of washout fluid, respectively.

A significant correlation was found between the UBM grade and the total number of droplets in the first 2 mL of washout fluid (*r* = 0.345, *P*=0.046, Spearman's correlation; Table 1). The number of smaller SO droplets (0.4–5 *μ*m) was also positively correlated to the UBM grade (*r* = 0.343, *P*=0.047), while the number of larger droplets (5–12 *μ*m) was not (*r* = 0.160, *P*=0.367; Table 1).

A similar correlation was found between the SOI obtained with B-scan ultrasonography and the total number of droplets in the last 2 mL (*r* = 0.342, *P*=0.048; Table 1). Again, the number of smaller SO droplets (0.4–5 *μ*m) was significantly correlated with the SOI (*r* = 0.358, *P*=0.037), while for the larger droplets (5–12 *μ*m), it was not (*r* = 0.052, *P*=0.771; Table 1).

## 4. Discussion

In this study, the possible correlations between the SO emulsification results evaluated by Coulter counter and UBM or B-scan ultrasonography were explored. Since UBM reflected the SO emulsification before SO removal and the first washout fluid were also collected before SO removal, the possible correlation between the two was explored. In the same way, B-scan reflected SO residual after SO removal, and the last washout fluid were also collected at the end of SO removal, which could also reflect SO residual. Therefore, correlation between the two were also studied. A significant correlation was found between the number of droplets and the quantitative assessment of SO emulsification provided by the imaging modalities. The main advantages of the latter are the high reproducibility and non-invasiveness; furthermore, these methods do not require any specific technique or software. On the other hand, the Coulter counter could simultaneously and accurately count and measure the size of SO droplets, though this method needs expensive equipment and SO samples.

To the best of our knowledge, this study reports the first direct comparison of these methods. Our findings were consistent, as both UBM and B-scan ultrasonography results were closely correlated with the total number of SO droplets measured with Coulter counter. This result was not granted since the resolution of UBM and B-scan ultrasonography is approximately 40 and 500 *μ*m, respectively, while the emulsified SO droplets are markedly below those dimensions. The emulsified SO droplets appear as highly reflective dots in ultrasound images; thus, these modalities can detect SO droplets of all sizes. This characteristic might explain the close correlation between the results of these scans and the total number of droplets identified with the Coulter counter.

Previous studies suggested a consistency between the results obtained with these methods. A significant negative correlation was found between age and SO emulsification evaluated by both UBM [16] and Coulter counter [21]. Furthermore, a close relationship between SO emulsification and elevated IOP was confirmed by studies using UBM [16, 24], B-scan ultrasonography [17], or Coulter counter [21]. These similarities between the results acquired with different methods support the close relationships between them. We also noticed that the UBM and B-scan ultrasonography results were significantly correlated with the number of small SO droplets and not with the larger ones, though the reason remains to be determined. However, this finding may be simply due to the small droplets being the vast majority (99.09 ± 0.82% and 98.22 ± 3.26% of the total in the first and last washout).

Several studies have been conducted to observe and evaluate SO emulsification, improving our understanding of this condition [19, 25]. The three methods we evaluated could all assess SO emulsification, from different perspectives. In addition to being performed at different time points and in different parts of the eye, B-scan ultrasonography and Coulter counter analysis study the droplets in the vitreous cavity, whereas UBM can also detect those infiltrating other tissues [22]. Previous studies also reported the differences and limitations of these methods [18, 19, 25]. Azzolini et al. [25] noted that, in UBM examinations, some SO droplets could be masked by after-ringing effects from other SO droplets. Moreover, the Coulter counter has a sensitivity between 0.4 and 30 *μ*m, and not all droplets were considered [19]. Thus, these methods have limitations; however, they could be considered complementary since they are performed at different time points and identify droplets in different parts of the eye. With the combination of their results, we could have a more complete view of SO emulsification, although first we should ensure that their results are comparable.

With this study, we verified the strong associations between the results of these three methods; therefore, we confirmed that the results from previous studies using different methods can be compared and evaluated together. Moreover, these three methods could all be used to assess SO emulsification in future research. Through a comprehensive analysis, a more thorough and complete knowledge of SO emulsification could be achieved, and subsequently, patient management could be improved. Among these methods, UBM could be used preoperatively and might be more relevant since the results could provide additional information before the SO removal procedure. Patients with severe emulsification identified by UBM should be monitored closely and receive a thorough irrigation of the vitreous cavity.

Our study was limited by its single-center, cross-sectional design and the limited number of cases.

## 5. Conclusion

UBM, Coulter counter, and B-scan ultrasonography provide reliable and highly consistent findings; these tests could all be used in the evaluation of SO emulsification, and their findings are comparable.

## Figures and Tables

**Table 1:** Correlations between number of SO droplets in the first or last washout and UBM total grade or SOI.

Number of droplets with a diameter of (*μ*m)	UBM total grade		SOI	
	*r* value	*P* value	*r* value	*P* value
0.4–<5	0.343	0.047^*∗*^	0.358	0.037^*∗*^
5–<12	0.160	0.367	0.052	0.771
0.4–<12	0.345	0.046^*∗*^	0.342	0.048^*∗*^

^*∗*^ *P* < 0.05 was considered statistically significant.

## Data Availability

The research data used to support the findings of this study are included within the article.

## References

[1] Cibis P. A., Becker B., Okun E., Canaan S. (1962). The use of liquid silicone in retinal detachment surgery. *Archives of Ophthalmology*.

[2] Ichhpujani P., Jindal A., Jay Katz L. (2009). Silicone oil induced glaucoma: a review. *Graefes Archive for Clinical and Experimental Ophthalmology*.

[3] Lewis H., Burke J. M., Abrams G. W., Aaberg T. M. (1988). Perisilicone proliferation after vitrectomy for proliferative vitreoretinopathy. *Ophthalmology*.

[4] Miller J. B., Papakostas T. D., Vavvas D. G. (2014). Complications of emulsified silicone oil after retinal detachment repair. *Seminars in Ophthalmology*.

[5] Abrams G. W., Azen S. P., Barr C. C. (1995). The incidence of corneal abnormalities in the silicone study. Silicone study report 7. *Archives of Ophthalmology*.

[6] Chen Y., Lam Ip Y., Zhou L. (2021). What is the cause of toxicity of silicone oil?. *Materials*.

[7] Scheerlinck L. M., Schellekens P. A., Liem A. T., Steijns D., van Leeuwen R. (2016). Incidence, risk factors, and clinical characteristics of unexplained visual loss after intraocular silicone oil for macula-on retinal detachment. *Retina*.

[8] Herbert E. N., Habib M., Steel D., Williamson T. H. (2006). Central scotoma associated with intraocular silicone oil tamponade develops before oil removal. *Graefes Archive for Clinical and Experimental Ophthalmology*.

[9] Taherian K., Bishop F., Woon W. H., Nelson M. (2004). Silicone oil endotamponade--is it safe?. *Eye*.

[10] Grzybowski A., Pieczynski J., Ascaso F. J. (2014). Neuronal complications of intravitreal silicone oil: an updated review. *Acta Ophthalmologica*.

[11] Fangtian D., Rongping D., Lin Z., Weihong Y. (2005). Migration of intraocular silicone into the cerebral ventricles. *American Journal of Ophthalmology*.

[12] Papp A., Toth J., Kerenyi T., Jackel M., Suveges I. (2004). Silicone oil in the subarachnoidal space--a possible route to the brain?. *Pathology, Research & Practice*.

[13] Federman J. L., Schubert H. D. (1988). Complications associated with the use of silicone oil in 150 eyes after retina-vitreous surgery. *Ophthalmology*.

[14] Valone J., McCarthy M. (1994). Emulsified anterior chamber silicone oil and glaucoma. *Ophthalmology*.

[15] Savion N., Alhalel A., Treister G., Bartov E. (1996). Role of blood components in ocular silicone oil emulsification. Studies on an in vitro model. *Investigative Ophthalmology & Visual Science*.

[16] Zhao H., Yu J., Zong Y., Jiang C., Zhu H., Xu G. (2021). Characteristics of silicone oil emulsification after vitrectomy for rhegmatogenous retinal detachment: an ultrasound biomicroscopy study. *Frontiers of Medicine*.

[17] Shiihara H., Terasaki H., Yoshihara N. (2016). Amount of residual silicone oil in vitreous cavity is significantly correlated with axial length. *Retina*.

[18] Shiihara H., Sakamoto T., Terasaki H. (2017). Effect of fluid-air exchange on reducing residual silicone oil after silicone oil removal. *Graefes Archive for Clinical and Experimental Ophthalmology*.

[19] Chan Y. K., Cheung N., Chan W. S., Wong D. (2015). Quantifying silicone oil emulsification in patients: are we only seeing the tip of the iceberg?. *Graefes Archive for Clinical and Experimental Ophthalmology*.

[20] Yu J., Zong Y., Tan Y., Jiang C., Xu G. (2020). Comparison of repeated fluid-air exchange and passive drainage for removing residual emulsified silicone oil droplets. *Journal of Ophthalmology*.

[21] Yu J., Zong Y., Jiang C., Zhu H., Deng G., Xu G. (2020). Silicone oil emulsification after vitrectomy for rhegmatogenous retinal detachment. *Journal of Ophthalmology*.

[22] Grigera D. E., Zambrano A., Cazon G. P., Cavanagh E., Girado S. G. (2000). Ultrasound biomicroscopy in silicone oil-filled eyes. *Retina*.

[23] Chan Y. K., Cheng H. C., Wu J. (2018). A perfluorobutylpentane (F4H5)-based solution for the removal of residual emulsified silicone oil. *Acta Ophthalmologica*.

[24] Avitabile T., Bonfiglio V., Cicero A., Torrisi B., Reibaldi A. (2002). Correlation between quantity of silicone oil emulsified in the anterior chamber and high pressure in vitrectomized eyes. *Retina*.

[25] Azzolini C., Pierro L., Codenotti M., Bandello F., Brancato R. (1996). Ultrasound biomicroscopy following the intraocular use of silicone oil. *International Ophthalmology*.

